# Hyperemesis Gravidarum in Undiagnosed Gitelman's Syndrome

**DOI:** 10.1155/2016/2407607

**Published:** 2016-08-07

**Authors:** Maria Czarina Acelajado, R. Michael Culpepper, Wilburn D. Bolton III

**Affiliations:** Department of Medicine, University of South Alabama, Mobile, AL 36617, USA

## Abstract

*Introduction*. Gitelman's syndrome (GS) is an autosomal recessive inherited defect in the thiazide-sensitive sodium-chloride cotransporter (NCCT) in the renal distal convoluted tubule. Physiologic changes of pregnancy promote renal potassium wasting, but serum potassium levels are kept in the physiologic range by increased levels of progesterone, which resist kaliuresis. In the presence of GS, this compensatory mechanism is easily overwhelmed, resulting in profound hypokalemia. We present a case of an 18-year-old primigravida with undiagnosed GS who presented with hyperemesis gravidarum in her 7th week of pregnancy. This report adds to the limited experience with GS in pregnancy as reported in literature and provides additional information on medical management that leads to successful maternal and fetal outcomes.

## 1. Introduction

Gitelman's syndrome (GS) is an autosomal recessive inherited defect in the thiazide-sensitive sodium-chloride cotransporter (NCCT) in the distal convoluted tubule. Defective sodium-chloride absorption at this site causes urinary wasting of sodium, chloride, potassium, and magnesium, resulting in hypokalemic metabolic alkalosis with normal or reduced blood pressure [1].

Most commonly, this defect arises from inactivating mutations in the solute carrier family 12, member 3, SLC12A3 gene localized on the 16q13 chromosome, which encodes the cotransporter. As many as 200 distinct NCCT mutants related to Gitelman's syndrome have been identified to date [2–4]. While considered to be a rare disease, it is one of the more common inherited renal tubular disorders. The estimated prevalence of GS is 1 in 40,000 and typically presents after 6 years of age with some cases presenting in adulthood [5].

The clinical picture of GS varies and some patients may be asymptomatic owing to heterozygosity. Studies attempting to identify a genotype-phenotype correlation have not been successful, given that there are multiple mutations and very few patients. In general, increasing numbers of mutations result in more severe disease, and male patients appear to be more markedly affected than female patients, who may experience later onset and milder symptoms [6].

Maintaining electrolyte levels in pregnant patients with GS can be quite difficult. Potassium homeostasis typically remains normal in pregnancy despite multiple physiologic changes that promote renal potassium wasting: volume expansion, increased renal blood flow, increased glomerular filtration rate (GFR), and activation of the renin-angiotensin-aldosterone axis. Serum potassium levels are kept at physiologic levels by increased levels of progesterone, which resist kaliuresis. This compensatory mechanism can be easily overwhelmed by maternal conditions such as gastrointestinal disorders, alcoholism, malnutrition, diabetes mellitus, and tubulointerstitial disorders and can lead to maternal hypokalemia. Nausea and vomiting in pregnancy, which are very common (occurring in about 70% of pregnant women [7]), can lead to severe hypokalemia in a patient with GS. Its extreme form, hyperemesis gravidarum, seen in 0.3 to 2% of all pregnancies, can lead to ketonuria, marked electrolyte abnormalities, and encephalopathy [7], even without concomitant GS. We present a case of an 18-year-old primigravida with hyperemesis gravidarum complicated by profound hypokalemia and hypomagnesemia that persisted even after vomiting episodes ceased, and that was refractory to parenteral electrolyte supplementation.

## 2. Case Report

Internal Medicine was consulted for refractory hypokalemia and hypomagnesemia in an 18-year-old primigravida who presented with nausea and vomiting for one week in her 7th week of pregnancy. On admission, her blood pressure was 107/69 mm Hg with a heart rate of 104/min and a temperature of 36.7°C. Abdominal findings did not reveal focal tenderness or guarding. There was no jaundice. Her initial chemistry panel revealed a serum potassium level of 2.3 mmol/L, a serum bicarbonate level of 32 mmol/L, and a serum magnesium level of 0.45 mmol/L. Nausea and vomiting were controlled by ondansetron and promethazine, but the electrolyte abnormalities persisted and remained critically low in spite of the resolution of hyperemesis. Initial treatment consisted of intravenous isotonic saline and parenteral potassium and magnesium supplementation. After a total of 380 mmol of potassium and 16 grams of magnesium have been given intravenously, potassium and magnesium levels did not rise past 2.8 mmol/L and 0.45 mmol/L, respectively.

Serum chemistries and spot urine electrolytes performed on day 7 of hospitalization showed the findings outlined in Table 1. The arterial pH of 7.51 and pCO_2_ of 41 mm Hg at a time the serum bicarbonate level was 32 mmol/L defined a primary metabolic alkalosis. While the patient was receiving intravenous isotonic saline, the plasma renin activity measured 50 *μ*g/L/hr and serum aldosterone level 2.09 nmol/L, the combination indicating a state of secondary hyperaldosteronism. The failure of saline infusion to suppress renin secretion after cessation of gastrointestinal fluid losses and at appropriate blood pressure for her age and state of pregnancy pointed to the need to identify a separate basis for renin-stimulated hyperaldosteronism. The absence of hypertension discounted consideration of a primary renin secreting tumor or of renin stimulation from a renovascular disease such as fibromuscular dysplasia.

The presence of severe hypokalemia with a serum potassium level of 2.6 mmol/L and an inappropriate elevation of the transtubular potassium gradient (TTKG; see Table 1) gave evidence of an ongoing and pathological effect of the elevated aldosterone levels to cause continued loss of potassium in the urine. The urine sodium greater than 100 mmol/L on two occasions could represent the effect of ongoing saline infusion and achievement of adequate extracellular volume expansion or of inappropriate sodium loss via a renal tubular defect in sodium reabsorption and conservation. The simultaneous hypomagnesemia made consideration of the latter explanation of a tubulopathy more likely and fit with the clinical characteristics of GS.

Treatment was begun with amiloride started at 5 mg/day and increased to 10 mg/day while continuing oral potassium and magnesium supplementation of 150 mmol/day and 4000 mg/day, respectively. On this regimen, levels of both electrolytes rose with a serum potassium of 3.3 mmol/L and serum magnesium 0.74 mmol/L at discharge from the hospital (day 15 of hospitalization).

This patient had several readmissions for recurrence of the nausea and vomiting during her first trimester of pregnancy, each associated with profound hypokalemia and hypomagnesemia, and management continued according to the plan outlined earlier. Apart from the nausea and emesis, she was relatively asymptomatic and denied muscle cramping, weakness, palpitations, or syncope. In the 38th week of gestation, she gave birth via vaginal delivery to a healthy baby girl weighing 2545 grams (5 lb, 9.8 oz), with APGAR scores of 8 and 9. No obstetric complications were noted.

## 3. Discussion

Gitelman's syndrome (GS) is an inherited hypokalemic salt-losing tubulopathy with secondary hyperaldosteronism that affects the thiazide-inhibitable sodium-chloride cotransporter (NCCT) of the distal tubule, making its presentation similar to that of treatment with thiazide diuretics. Defects in the NCCT lead to increased delivery of sodium to the distal tubule, causing increased sodium reabsorption in exchange for potassium and hydrogen ions, potentially causing hypokalemia and metabolic alkalosis. It is important to differentiate GS from classic Bartter's syndrome which is also characterized by hypokalemia, renal potassium wasting, and activation of the renin-angiotensin-aldosterone axis [8]. Unlike Bartter's syndrome which affects the Na-K-2Cl transporter of the thick ascending limb and is manifest in infancy, patients with GS develop hypomagnesemia from increased urinary magnesium losses and have decreased urinary calcium excretion. Gitelman's syndrome typically has a good prognosis and a milder course than Bartter's and typically presents later in childhood or even in early adulthood.

During normal pregnancy, there is renal tubular loss of potassium and magnesium; this urinary potassium wasting occurs due to increased aldosterone levels and other substances with mineralocorticoid activity. In spite of this, serum potassium levels are kept at physiologic range because increased levels of progesterone resist kaliuresis. In a patient with GS, this mechanism may not fully compensate for the increased urinary losses of magnesium and potassium [9]. Gestational emesis and fetal demands for these electrolytes can further aggravate the clinical picture, as seen in this patient.

The required supplemental magnesium and potassium during the pregnancy in a patient with GS can be quite substantial. McCarthy and colleagues reported that a patient with GS required a total of 3,680 mmol of potassium and 940 mmol of magnesium supplementation given intravenously during the course of her pregnancy [10]. These estimates are reflected in the requirements in this patient of 80–160 mmol of potassium and 4 grams (150 mMol) of magnesium daily needed to make modest improvements in her electrolyte deficits.

It appears from the experience of others that the goal of management is not to achieve normal levels of potassium and magnesium; rather, it is to achieve levels at which the patient is relatively asymptomatic. Basu et al. described a patient with known GS who had three successful normal pregnancies in spite of low potassium and magnesium levels, with the potassium level during pregnancy remaining at 2.8–3.1 mmol/L [11]. Likewise, Talaulikar and Falk reported a case in a pregnant patient with GS, where the potassium and magnesium requirements increased sixfold during the pregnancy and normal levels of both electrolytes were not achieved despite intravenous supplementation [12]. In both cases, there was no adverse impact on the course of pregnancy or fetal outcome.

The cornerstone of therapy is oral supplementation of potassium and magnesium, and intravenous therapy may be considered if the patient cannot tolerate oral intake. Kwan and Falk described their experience in treating a pregnant patient with GS using just oral supplementation (potassium chloride 24 g/day and magnesium aspartate 16 g/day) and reported that their patient did not suffer from any adverse outcome [13]. Magnesium and potassium levels were maintained at the lower end of normal range.

The use of mineralocorticoid antagonists should be reserved for highly refractory cases, as its administration during pregnancy has been controversial for safety reasons. Although there is limited experience with the use of spironolactone or eplerenone in pregnancy, available data suggests these drugs are safe [14, 15]. Concern for decreased virilization in male infants of mothers who took spironolactone during pregnancy has been raised but was not the case in a patient described by de Arriba and colleagues [15]. Amiloride, which is a potassium-sparing diuretic that acts as a direct antagonist of the epithelial sodium channel (ENaC) in the distal convoluted tubules, has also been successfully used in pregnancy without report of any adverse outcomes [16].

The most common fetal complication seen in the case reports we reviewed was oligohydramnios, which could be related to the salt wasting seen in GS and the inability to maintain extracellular fluid volume [9, 10, 17]. McCarthy and colleagues recommend that in addition to monitoring serum electrolyte levels, determining maternal weight and volume status is imperative as well [10]. de Haan et al. also described prolonged labor that could be attributed to low serum potassium levels [9]. Women with GS have had both uneventful vaginal and caesarean deliveries and GS does not seem to predispose to the risk of premature delivery. In all reports, the infant did well after delivery.

To our knowledge, this is the first report of GS diagnosed during pregnancy complicated by hyperemesis gravidarum. The clue to the diagnosis was the difficulty in correcting magnesium and potassium levels in spite of massive amounts of intravenous and oral supplementation despite resolution of emesis and restoration of extracellular fluid volume with saline. While this patient was relatively asymptomatic leading up to her pregnancy, the diagnosis was unmasked by intractable vomiting episodes associated with the hyperemesis gravidarum. Her presentation highlights the milder course of GS compared to Bartter's and the possibility that the diagnosis may become evident only in early adulthood. Further, based on our experience, the use of amiloride during pregnancy was not associated with any adverse effects on the mother or the fetus.

## 4. Conclusion

GS is a rare inherited condition of the distal convoluted tubule that leads to hypokalemia and metabolic alkalosis. In normal pregnancy, there is an increased tendency towards kaliuresis, which is countered by excess progesterone. The occurrence of increased gastrointestinal losses as seen in the patient we described may aggravate the hypokalemia and hypomagnesemia already found in patients with GS. In an undiagnosed patient, the workup should include measurement of urine electrolytes, arterial blood gas, and plasma renin and aldosterone levels. The goals of treatment are to keep the patient normovolemic and asymptomatic, even if normal levels of potassium and magnesium are not attained. The use of mineralocorticoid antagonists and amiloride has been described and has not been associated with adverse effects on the mother and the fetus. There is limited experience reported in literature on GS in pregnancy. Available data suggest that GS is not associated with adverse effects on the pregnancy outcome.

## Figures and Tables

**Table 1:** Laboratory work-up. Summary of important laboratory tests performed during the patient's hospitalization. On day 6 of hospitalization, the calculated transtubular potassium gradient (TTKG) is 30.77, consistent with renal potassium wasting.

Parameter	On admission	6th hospital day	After amiloride 5 mg/day	After amiloride 10 mg/day	At the time of delivery
Creatinine (umol/L)	63.6	38.9	47.7	38	50.3
Blood urea nitrogen (mmol/L)	5	1.43	5.71	5	4.29
Potassium (mmol/L)	2.3	2.6	2.6 to 3.3	2.8 to 3.5	3
Sodium (mmol/L)	137	134	135	132	137
Magnesium (mmol/L)	0.45	0.45	0.58 to 0.74	0.58 to 0.82	0.62
Calcium (mmol/L)	2.33	2.2	2.33	2.33	2.15
HCO_3_ (mmol/L)	32	33	26	29	26
Chloride (mmol/L)	93	95	96	99	99
Serum osmolality (mOsm/kg)	—	286	—	—	—
Urine osmolality (mOsm/kg)	—	379	—	—	—
Spot urine chloride (mmol/L)	—	239	—	—	—
Spot urine sodium (mmol/L)	—	178	—	—	—
Spot urine creatinine (umol/L)	—	3978	—	—	—
Spot urine potassium (mmol/L)	—	106	—	—	—
pH	—	7.51	—	—	—
pCO_2_ (mm Hg)	—	41	—	—	—
